# Effects of long-term irrigation on soil phosphorus fractions and microbial communities in *Populus euphratica* plantations

**DOI:** 10.48130/FR-2023-0017

**Published:** 2023-07-26

**Authors:** Yue He, Xiazhen Lin, Lei Wang, Xiaomin Ma, Lidong Fang, Zhichao Xia

**Affiliations:** 1 School of Forestry & Landscape Architecture, Anhui Agricultural University, Hefei 230036, China; 2 Teaching Center, Zhejiang Open University, Hangzhou 310012, China

**Keywords:** Irrigation, Phosphorus (P) fractions, P cycling, Soil microbial properties, Alkaline phosphatase gene communities

## Abstract

Irrigation has been demonstrated to be effective in managing *Populus euphratica* plantations, but its impacts on phosphorus (P) availability and the soil microbiome have not been fully elucidated. In this study, we compared soil properties, P fractions, phosphatase activities, and microbial communities in the surface soil (0–20 cm) of *P. euphratica* plantations under both drought and irrigation conditions. We found that total P, labile P and moderately labile P all increased significantly under irrigation by 12.3%, 70.1%, and 3.0%, respectively. The increased levels of labile P were primarily driven by higher levels of NaHCO_3_-Pi, which increased from 1.9 to 12.3 mg·kg^−1^. Furthermore, irrigation markedly altered labile P composition and the relative levels of resin P, NaHCO_3_-Pi, and NaHCO_3_-Po were all impacted. Improved soil moisture increased soil phosphatase activity, suggesting that soil organic P (Po) mineralization was positively affected by irrigation. Moreover, we observed that bacterial diversity, fungal diversity, and alkaline phosphatase gene communities, rather than total microbial biomass carbon or total phospholipid fatty acids, were most explained in the dynamics of soil P fractions. Furthermore, we found positive correlations among inorganic P (Pi) and Bradyrhizobiaceae, Nocardiaceae, and Sphingomonadaceae, whereas negative correlations were found between Burkholderiaceae and Pi, highlighting the diverse functional bacteria involved in P cycling. Our study demonstrates that irrigation can increase soil P availability and supply capacity, with shifts in P composition closely linked to changes in soil microbial characteristics. Water management strategies that target the restoration of soil microbial communities may therefore improve soil quality and enhance soil P cycling.

## Introduction

Ecologically vulnerable regions are more susceptible to the impacts of global climate change^[1]^. Extreme droughts are likely to be more frequent in arid regions^[2]^, and soil moisture is a critical limiting factor for many ecosystems. Soil properties and microbiomes are detrimentally affected by drought, leading to changes in pH levels, organic matter content, microbial diversity, and nutrient dynamics, with the essential macronutrient phosphorus (P) often seriously impacted^[3−6]^. P enters the soil *via* weathering of P-bearing primary minerals. Following release into the soil, P undergoes complex geochemical and biological transformations^[7]^, resulting in a diversity of coexisting organic and inorganic forms. The turnover rates and bioavailability of these various forms of P to plants and microbes vary significantly in the soil^[8]^. Despite studies indicating that increasing frequency of droughts will profoundly impact soil P cycling, few studies fully consider soil P cycling in ongoing drought manipulation experiments in forest ecosystems.

Plants assimilate P from the soil *via* their root systems. Root exudates, such as organic acids and phosphatase enzymes, facilitate the solubilization and mobilization of soil P^[9]^, and root architecture significantly influences P uptake efficiency^[9]^. Organic P (Po) in the soil is predominantly mineralized by plant roots and soil microorganisms, which produce a variety of enzymes that impact this process, primarily extracellular acid phosphatases (ACPs) and alkaline phosphatases (ALPs)^[10]^. While plant roots primarily produce ACPs, soil microorganisms, particularly bacteria, are the primary producers of ALPs^[11]^. Soil ACP and ALP activities have been shown to be directly dependent on soil water availability^[12]^. Drought can alter the composition and function of microbial communities in the soil, thereby influencing soil nutrient cycling^[13]^.

The relationship between soil P fractions and microbial communities remains unclear and largely depends on which aspects of microbial communities are evaluated. Microbial groups, such as bacteria and fungi, display highly variable capacity to utilize soil P^[14]^. The abundance, composition, and functional diversity of soil microorganisms can be assessed using microbial biomass (measured through phospholipid fatty acid [PLFA] biomarkers and microbial biomass carbon [MBC]), composition and diversity of taxonomic communities (taxonomic profiles measured using 16S rRNA or ITS genes and their amplicon sequencing or PLFA biomarkers), or potential functions (profiles of functional genes measured using qPCR). Secreted ALP activity is primarily driven by *phoD* and *phoX*^[10,15]^. ALP activity and the prevalence of the *phoD* gene have also been shown to be directly correlated^[16]^, and the abundance and diversity of the *phoD* genes are affected by soil pH^[17]^ as well as fertilizer inputs^[18]^. However, research aimed at explicitly elucidating the association between microbial communities and soil P components is still lacking. Clarifying these relationships will be beneficial to integrating soil microbial processes into soil P cycling models.

*Populus euphratica* trees play a crucial role in desert ecosystems but are susceptible to the impacts of global climate change. As a dominant tree species in arid areas, *P. euphratica* can be used for windbreaking, sand fixation, and soil water conservation^[19]^. It provides multiple ecosystem services as a natural barrier to the expansion of deserts^[20]^. Given the projected increase in the frequency and severity of droughts, *P. euphratica* trees will likely face increasing environmental stresses^[9,21−23]^. The Chinese government has implemented projects aimed at restoring *P. euphratica* populations, including groundwater irrigation near the Tarim River, which has successfully facilitated the recovery of *P. euphratica*^[24]^. Nevertheless, the impacts of long-term irrigation on soil P cycling processes in *P. euphratica* plantations, along with the relationships between soil P fractions and soil microorganisms, are currently unknown. In this study, we assessed the impacts of irrigation on soil P pools and the soil microbiome to test three hypotheses: (1) irrigation significantly affects soil P status, particularly labile P fractions; (2) irrigation significantly increases soil phosphatase activity, an outcome that may be correlated with soil properties and microbial changes; (3) the association between P fractions and soil microbes may depend on the metrics used to evaluate microbiome characteristics.

## Materials and methods

### Field sites

This study was performed on the northwestern border of the Tarim Basin, located in China's Xinjiang Autonomous Region. The climate in the region is characterized by an annual mean air temperature of 10.8 °C and a mean annual precipitation of 50 mm. The soil type in the area is classified as calcic xerosol.

A factorial experiment was conducted using two water management conditions, with six replicates each. A total of 12 plots, each measuring 15 m × 10 m, were selected along the upper reaches of the Tarim River (81°17′ E, 40°32′N – 40°81′ N). *Populus euphratica* trees were planted in 2003 as part of a vegetation restoration project.

For the initial five years, all plots received irrigation. From 2009 to 2021, half of the plots were randomly selected to be irrigated for half a month in March and April (irrigation treatment), while the other half only received ambient precipitation (drought treatment). Irrigation was maintained for eight hours per day at approximately 50 m^3^·h^−1^, and the relative soil moisture content was kept at 90% to a depth of 60 cm during the irrigation period^[9]^.

### Soil sampling

Six 2 m × 2 m sub-plots were randomly selected for each water treatment to ensure representative sampling. In mid-August 2021, three samples were collected from the top 20 cm of soil in each plot and combined to form six composite samples. The composite samples were placed in sterile sealed bags and kept on ice during transportation to the laboratory for processing. Subsequently, the samples were thoroughly mixed and passed through a 2-mm sieve. Portions of each fresh soil sample were stored at 4 °C and −80 °C for further analysis, while the remaining portion was air-dried and divided into an archival sample and a sample used to determine soil properties and P fractions.

### Soil properties

Soil pH was determined using a pH meter, with a soil-to-CaCl_2_ solution ratio of 1:2.5 (v/v), following standard protocols. Soil organic carbon (SOC) was quantified using the electric sand bath potassium dichromate titration method, as described by Bremner and Jenkinson^[25]^. Total nitrogen (TN) content was measured using the micro-Kjeldahl method. ${\text{NH}^+_4} $-N and ${\text{NO}^-_3} $-N concentrations were extracted from the soil using 1M KCl at a ratio of 1:5 and analyzed using a Continuous-Flow Auto Analyzer (Bran+Luebbe AA3, Germany). Exchangeable K^+^, Na^+^, Ca^2+^, and Mg^2+^ were extracted using acetamide and analyzed using an inductively coupled plasma emission spectrometer (ICP).

### Soil P fractions

Soil P was fractionated using the continuous extraction method originally developed by Hedley et al.^[26]^ and modified by Tiessen^[27]^. The following protocol was used to extract P fractions from 0.5 g of air-dried soil deposited in a 50 mL centrifuge tube: (a) each centrifuge tube received two 9 mm × 62 mm resin strips along with 30 mL distilled water, followed by stirring at 160 rpm for 16 h to extract P from the resin strips using 0.5 M HCl (referred to as resin-Pi); (b) following the removal of the aqueous solution, 30 mL of 0.5 M NaHCO_3_ at pH 8.5 was added and the tubes were shaken for 16 h to extract NaHCO_3_-P; (c) NaOH-P was extracted by adding 30 mL of 0.1 M NaOH and rotating the tubes for 16 h; (d) 30 mL of 1 M HCl was added to each centrifuge tube, and the tubes were shaken for 16 h to extract 1 M HCl-Pi; (e) 15 mL of concentrated HCl was used to further extract soil residue at 80 °C (conc. HCl-P); (f) P was obtained by boiling the soil residue in 8 mL of concentrated H_2_SO_4_ with 10 drops of HClO_4_, to obtain residual P.

After extraction, the supernatant was partitioned into two aliquots for Pi and Po determination. The quantification of Pi was carried out using the molybdate-ascorbic acid procedure originally proposed by Murphy & Riley^[28]^. Total P (TP) was determined by incubating the supernatant with acidified ammonium persulfate at 121 °C for 1 h. TP and Pi were directly measured from the extracts, and Po was calculated by subtracting TP from Pi^[29]^. To determine soil microbial biomass P (MBP), chloroform fumigation and NaHCO_3_ extraction were carried out over a 24-h period, as described by Brookes et al.^[30]^. Separate non-fumigated samples were spiked with 25 mg·L^−1^ of P to evaluate the efficacy of P recovery during the fumigation process.

### Activities of ACP and ALP

ACP and ALP activities in soil samples were determined *via* the method described by Tabatabai^[31]^. Briefly, fresh soil samples (1 g) were incubated at pH 6.5 (for ACP) and pH 11.0 (for ALP) at 37 °C for 1 h, using p-nitrophenyl phosphate (pNPP) and disodium phenyl phosphate as substrates. ACP and ALP activities were recorded as mg p-nitrophenol and phenol·kg^−1^ soil (dry weight)·h^−1^, respectively.

### Soil microbial characteristics

We determined microbial biomass through analysis of MBC and PLFAs. Extraction of MBC was performed by adding 0.5 M K_2_SO_4_ to both chloroform-fumigated and unfumigated soil samples, followed by measurement with a computerized total organic carbon analyzer (Analytikjena, Germany). MBC was quantified by calculating the variation in organic carbon extracted between the fumigated and unfumigated soils. PLFAs were obtained from freeze-dried soil (5 g) following the procedure described by Frostegård et al.^[32]^. PLFAs were further classified into respective microbial functional groups in accordance with the procedure created by Ruess & Chamberlain^[33]^. Total PLFAs were determined by summing the biomass of all microbial functional groups. The bacterial to fungal ratio (B: F) was calculated by dividing the sum of all bacterial biomarkers by the sum of all fungal biomarkers.

Soil DNA was extracted from freeze-dried soil samples using the PowerSoil® DNA Isolation Kit (MO BIO Laboratories Inc., Carlsbad, CA, US), following the manufacturer's instructions. Amplifications of bacterial 16S (V4-V5) and fungal ITS rRNA genes, as well as quantitative polymerase chain reaction (qPCR) and amplicon sequencing of *phoD* and *phoX*, were performed following the protocols described by Xia et al.^[34]^ and are provided in Supplemental Material S1.

### Statistical analysis

T-tests were employed to determine variations in soil properties, P fractions, ACP and ALP, total PLFAs, MBC, MBP, bacterial and fungal Shannon-diversity, copy numbers of *phoD* and *phoX* genes, and alpha diversity (richness and Shannon-diversity) of alkaline phosphatase gene communities between the drought and irrigation treatment groups. The Wilcoxon test was used to detect variations in the relative abundance of *phoD* and *phoX* bacteria at the family and genus levels between the two treatments. The 'ggvegan' R package was used to conduct a covariance analysis for soil properties and P fractions. To investigate the associations between soil properties and soil P fractions, as well as between soil microbial characteristics and soil P fractions, we employed redundancy analysis (RDA). Additionally, the 'vegan' R package was employed to conduct principal co-ordinates analysis (PCoA) for *phoD* and *phoX* community composition and procrustean analysis among the *phoD* and *phoX* community with soil P fractions. The correlations between soil P fractions with the *phoD* and *phoX* community, as well as MBP, ACP, and ALP, were performed using the 'psych' and 'pheatmap' R packages. All box plots were generated utilizing the 'ggplot2' package in R (https://cran.r-project.org/package=ggplot2).

## Results

### Soil properties, P fractions, and their associations

**Figure 1:**
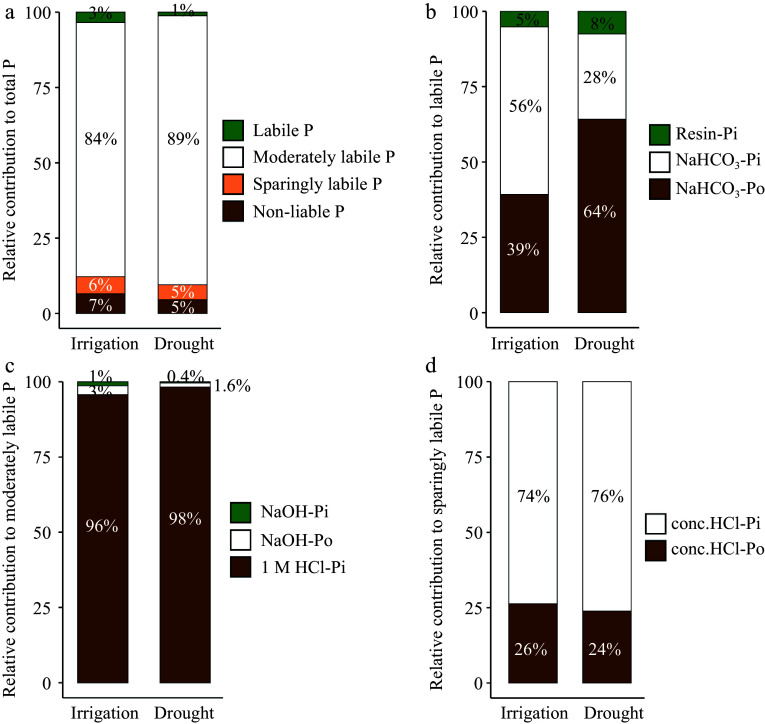
Percentage of each phosphorus (P) fraction under different water management treatments. (a) Total P. (b) Labile P. (c) Moderately labile P. (d) Sparingly labile P.

Compared to the drought treatment, irrigation significantly increased several soil physical and chemical attributes, including WC, pH, TN, SOC, ${\text{NH}^+_4} $-N, TP, and TPi (Table 1). The labile P fraction represented approximately 1%−3% of TP, while the moderately labile P fraction accounted for 84%−89% and decreased under irrigation (Fig. 1a). The relative content of resin-Pi decreased slightly, whereas the amount of NaHCO_3_-Pi in the labile P fraction increased significantly with irrigation (Fig. 1b). The NaHCO_3_-Pi fraction accounted for approximately 56% and 28% of labile P under irrigation and drought treatments, respectively (Fig. 1b). The components comprising moderately labile P were minimally altered by irrigation (Fig. 1a). The ratio of NaOH-extracted P in the moderately labile P fraction increased by 2% comparing drought to irrigation treatment, while the fraction of 1 M HCl-Pi was reduced by 2% (Fig. 1c). Irrigation led to a slight variation in the relative concentration of conc. HCl-Pi, which varied between 74%−76% under the two water treatments (Fig. 1d).

**Table 1:** Effects of irrigation on soil properties (mean ± standard error) in *P. euphratica* plantations.

Soil properties	Irrigation	Drought	*p* value
WC (%)	25.26 ± 0.54	6.93 ± 0.95	**< 0.01**
pH	8.41 ± 0.05	8.72 ± 0.02	**< 0.01**
TN (g·kg^−1^)	1.18 ± 0.05	0.76 ± 0.02	**0.03**
SOC (g·kg^−1^)	40.13 ± 0.22	32.12 ± 0.29	**< 0.01**
$ {\text{NO}^-_3}$-N (mg·kg^−1^)	6.35 ± 1.82	4.55 ± 0.90	0.16
${\text{NH}^+_4} $-N (mg·kg^−1^)	2.15 ± 0.23	1.25 ± 0.11	**< 0.01**
TP (g kg^−1^)	0.65 ± 0.01	0.57 ± 0.01	**0.01**
DON (mg·kg^−1^)	7.96 ± 0.47	9.65 ± 0.27	0.05
AK (g·kg^−1^)	0.41 ± 0.09	0.32 ± 0.05	0.12
DOC (g·kg^−1^)	0.26 ± 0.03	0.39 ± 0.07	0.17
Na^+^ (g·kg^−1^)	1.85 ± 0.06	1.82 ± 0.16	0.37
Ca^2+^ (g·kg^−1^)	15.75 ± 0.51	13.95 ± 0.34	0.86
Mg^2+^ (g·kg^−1^)	0.83 ± 0.08	0.61 ± 0.03	0.06
TPi (g·kg^−1^)	0.57 ± 0.01	0.53 ± 0.01	**0.01**
Pi/Pt (%)	88.15 ± 0.91	92.29 ± 0.30	0.68
Po/Pt (%)	11.85 ± 0.91	7.71 ± 0.30	0.16
Bold numbers indicate significant differences (*p* < 0.05) between treatments. WC: water content, TN: total nitrogen, SOC: soil organic carbon, DON: dissolved organic nitrogen, AK: available K, DOC: dissolved organic carbon. Total P is the sum of all P fractions; total Pi is the sum of Resin-Pi, NaHCO_3_-Pi, NaOH-Pi, 1 M HCl-Pi, and conc. HCl-Pi; total Po is the sum of NaHCO_3_-Po, NaOH-Po, and conc. HCl-Po.			

The concentrations of labile and moderately labile P increased markedly under irrigation (Table 2). The Pi and Po fractions in the labile P and moderately labile P fractions also increased under irrigation. Although sparingly labile P values were not significantly different between irrigation and drought treatments, irrigation had significantly higher conc. HCl-Po and conc. HCl-Pi than the drought treatment (Table 2).

**Table 2:** Soil phosphorus (P) sequential fractionation under different water management treatments.

P fraction (mg·kg^−1^)	Irrigation	Drought	*p* value
Labile P			
Resin-Pi	1.15 ± 0.27	0.50 ± 0.06	**0.04**
NaHCO_3_-Pi	12.31 ± 2.40	1.88 ± 0.51	**< 0.01**
NaHCO_3_-Po	8.69 ± 0.82	4.25 ± 0.49	**0.03**
ΣLabile P	22.15 ± 1.67	6.62 ± 0.54	**< 0.01**
Moderately labile P			
NaOH-Pi	7.02 ± 0.79	2.07 ± 0.12	**< 0.01**
NaOH-Po	16.41 ± 1.83	6.98 ± 0.17	**< 0.01**
1 M HCl-Pi	521.10 ± 12.37	505.52 ± 1.56	**0.03**
ΣModerately labile P	544.53 ± 13.51	511.58 ± 1.54	**0.02**
Sparingly labile P			
Conc. HCl-Pi	27.50 ± 1.84	21.35 ± 1.73	**0.01**
Conc. HCl-Po	9.79 ± 1.38	6.68 ± 1.50	**0.02**
ΣSparingly labile P	37.29 ± 2.75	28.04 ± 1.95	0.17
Nonlabile P			
Residual P	41.76 ± 5.11	26.22 ± 0.82	0.49
Bold numbers indicate significant differences (*p* < 0.05) between treatments.			

Redundancy analysis (RDA) identified variations in soil P fractions between drought and irrigation (Supplemental Fig. S1a), and soil properties explained almost 98% of the variation observed in soil P fractions (Supplemental Fig. S1a). Of the soil physicochemical parameters evaluated, WC and pH had the most explanatory power for the observed variations in soil P fractions. Except for 1M HCl Pi, all soil P parameters were positively correlated with higher WC, ${\text{NH}^+_4} $-N, and AK and negatively associated with lower pH values (Supplemental Fig. S1b).

### Soil phosphatase enzymes and microbial characteristics

**Figure 2:**
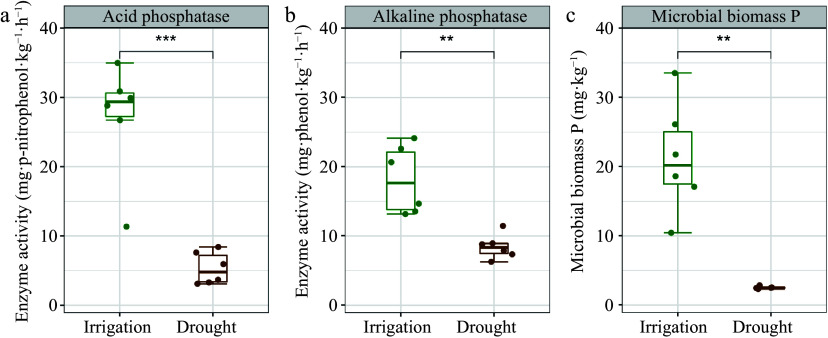
Soil phosphatase and microbial biomass P in *P. euphratica* plantations under different water management treatments. (a) Acid phosphatase activity. (b) Alkaline phosphatase activity. (c) Microbial biomass P. The error bars indicate the SE of the mean (n = 6). Asterisks indicate the level of significance: ** *p* < 0.01, *** *p* < 0.001.

**Figure 3:**
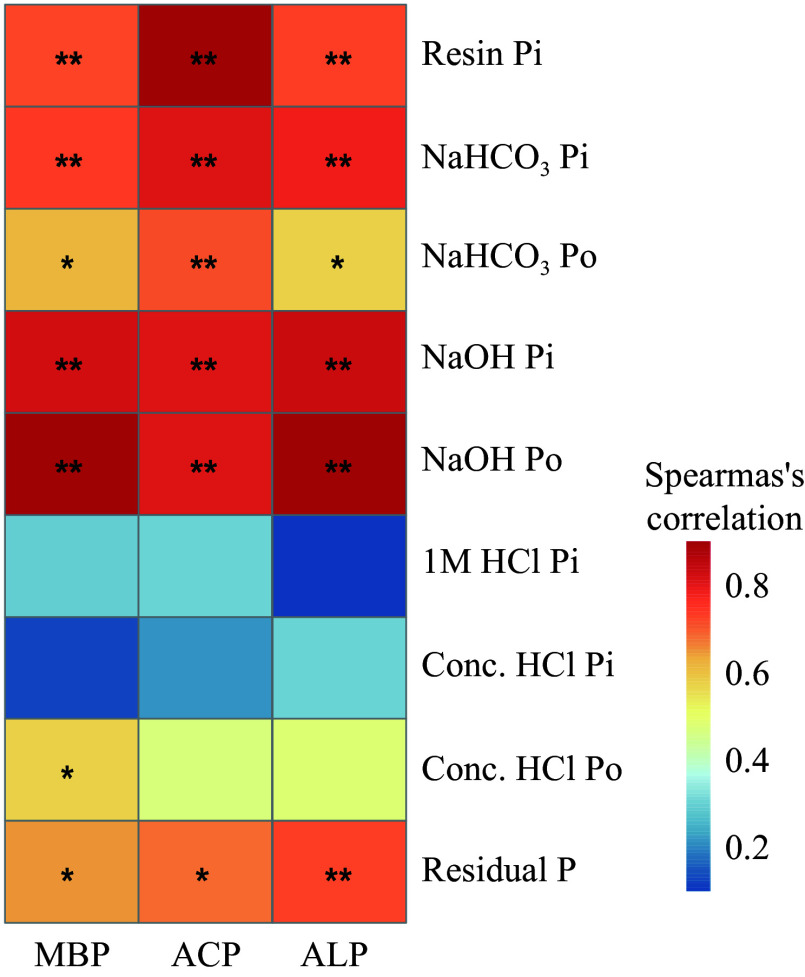
Spearman's correlation analysis among soil P fractions and soil phosphatase (acid and alkaline phosphatase) activity and microbial biomass P. Significance of changes in each P fraction: * *p* < 0.05; ** *p* < 0.01.

**Figure 4:**
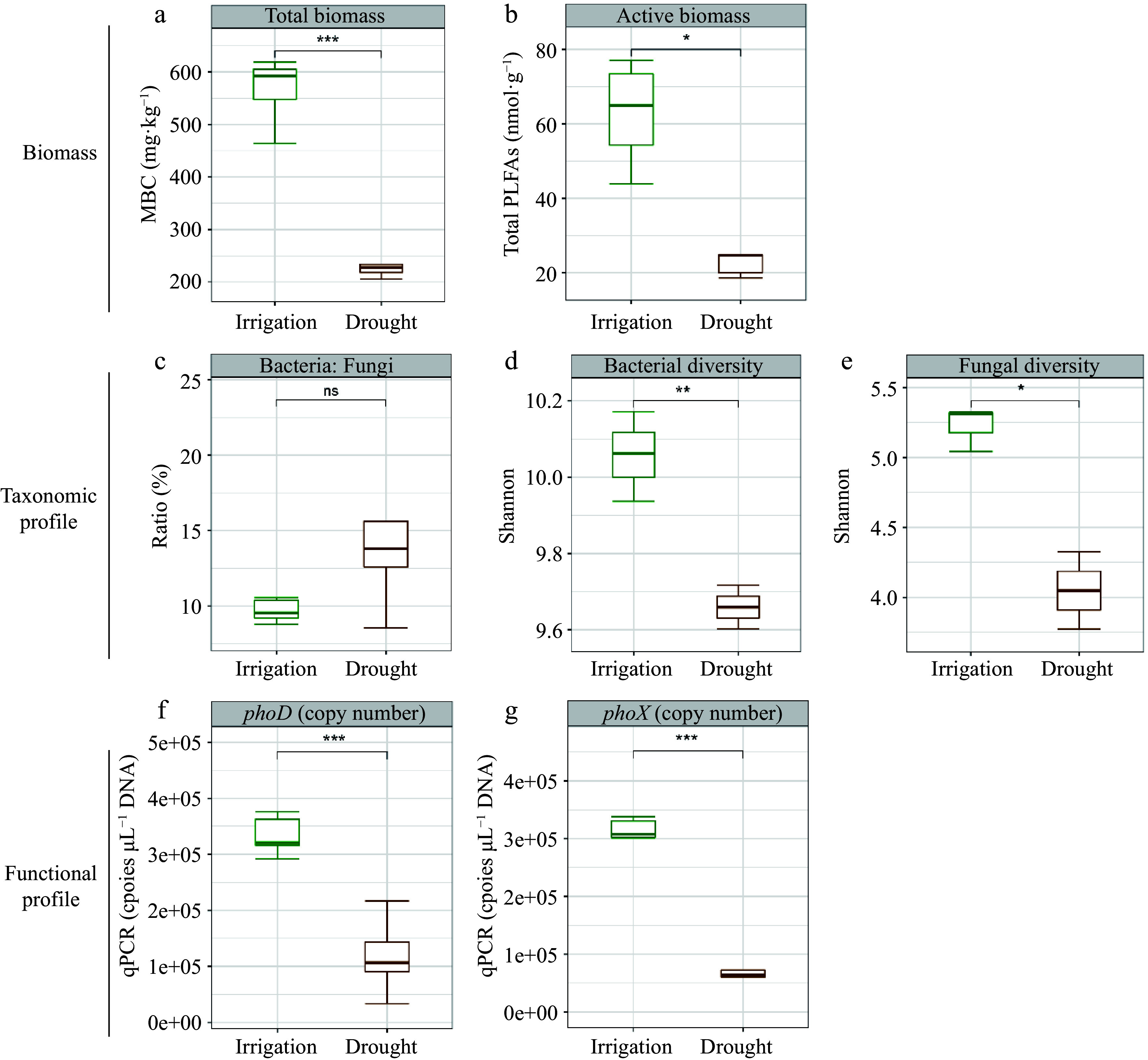
Effects of water management on soil microbial characteristics (biomass change, taxonomic profile and functional profile). (a) Total microbial biomass C. (b) Total PLFAs. (c) Bacteria to fungi ratio. (d) Shannon diversity of bacteria. (e) Shannon diversity of fungi. (f) *phoD* copies. (g) *phoX* copies. Significance levels were standardized across the panels (* *p* < 0.05; ** *p* < 0.01 and *** *p* < 0.001).

Under irrigation, there was a significant increase in MBP, ACP, and ALP relative to the drought treatment (Fig. 2a−c). Notably, irrigation increased ACP more strongly than ALP (Fig. 2a & b). Significant and positive relationships were identified between labile P and moderately labile P, as well as between residual P and MBP, ACP, and ALP. However, HCl-P did not exhibit noteworthy associations with MBP, ACP, or ALP, with the exception of MBP and conc. HCl Po (Fig. 3). To assess the impact of water management on soil microbial characteristics, microbial analyses were conducted using three metrics: biomass changes, taxonomic profiles, and functional changes (Fig. 4a−g). This analysis showed that soil microbial biomass was greater under irrigation than under drought, regardless of whether it was measured using MBC or total PLFAs (Fig. 4a & b). While there were no substantial differences between the two treatments in the relative proportions of bacteria and fungi based on PLFA classification, the diversity of bacteria and fungi under irrigation was higher than under drought when it was assessed using bacterial 16S and fungal ITS rRNA gene amplifications (Fig. 4c−e). Additionally, *phoD* and *phoX* copy numbers were higher under irrigation than under drought (Fig. 4f & g).

**Figure 5:**
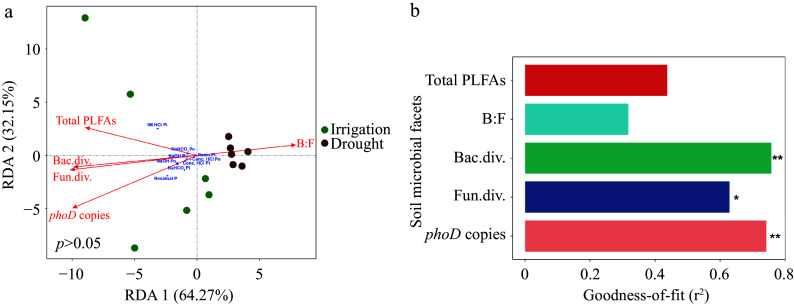
Redundancy analysis of soil P fractions impacted by soil microbial characteristics. (a) RDA across all experimental units. (b) The variation in soil microbial characteristics explaining soil P fractions. Red arrows represent soil microbial characteristics. Blue crosses represent soil P fractions. Significance is indicated by ** *p* < 0.01; * *p* < 0.05.

Notably, all soil microbial parameters except the bacteria-to-fungi ratio were positively correlated with elevated levels of soil P (Fig. 5a). Among the evaluated soil microbial characteristics, microorganism composition and functional levels exhibited better explanatory power for the variations in soil P fractions (Fig. 5b).

The richness and diversity of the bacterial *phoD* genes were significantly higher under irrigation than under drought (Supplemental Fig. S2a & b). In contrast, no major variations were observed between treatments in the richness and diversity of bacterial *phoX* genes (Supplemental Fig. S2b). Principal coordinate analysis (PCoA), conducted using the Bray-Curtis distance matrix, indicated significant variations in *phoD* and *phoX* gene communities between treatments (Supplemental Fig. S2c & d).

The taxonomic composition of *phoD* and *phoX* gene bacterial communities was assessed at the family and genus levels, where the relative abundances exceeded 0.01% (Supplemental Fig. S3a−d). Specifically, *phoD* gene reads were primarily classified into 14 families and 14 genera, while *phoX* gene reads were classified into 14 families and 13 genera. Analysis of *phoD* gene community composition at the family level revealed that irrigation had a considerable impact on the relative abundance of Bradyrhizobiaceae, Nocardiaceae, Sphingomonadaceae, and Burkholderiaceae (Supplemental Fig. S3a). Furthermore, at the genus level, irrigation increased the relative abundance of *Bradyrhizobium* and *Rhodococcus* (Supplemental Fig. S3b). Similarly, *phoX* gene community composition analysis demonstrated that irrigation significantly affected the relative abundance of Phyllobacteriaceae and Xanthomonadaceae at the family level. In contrast, at the genus level, the relative abundance of *Halomonas* and *Rhodopirellula* reduced and increased during irrigation, respectively (Supplemental Fig. S3c & d).

**Figure 6:**
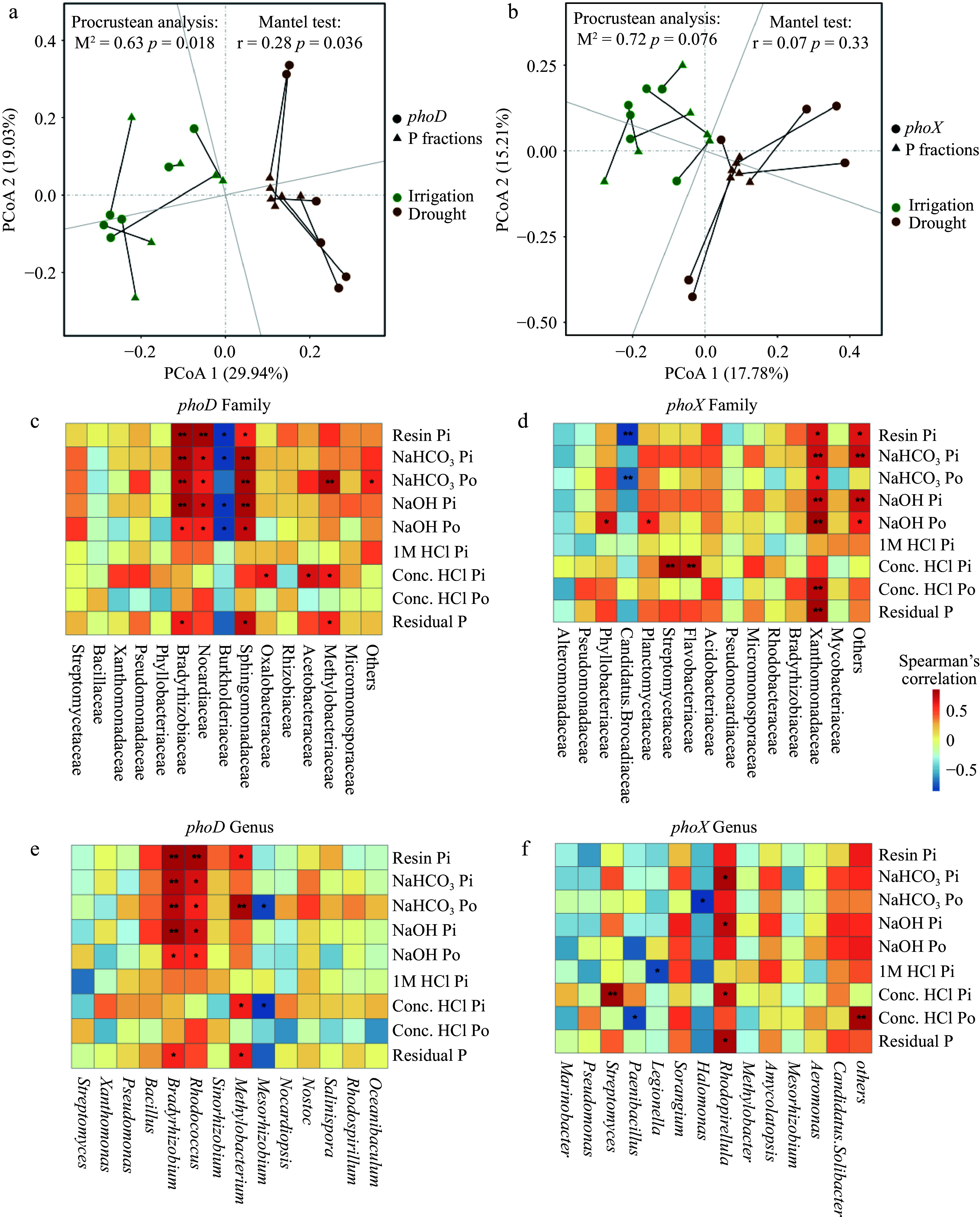
Procrustean analyses and spearman's correlations between soil P fractions and alkaline phosphatase gene communities. (a) Procrustean analyses of *phoD* community composition and soil P fractions across samples. (b) Procrustean analyses of *phoX* community composition and soil P fractions across samples. (c) Spearman’s correlations of soil P fractions and the relative abundance of *phoD* at family level. (d) Spearman's correlations of soil P fractions and the relative abundance of *phoX* at family level. (e) Spearman's correlations of soil P fractions and the relative abundance of *phoD* at genus level. (f) Spearman's correlations of soil P fractions and the relative abundance of *phoX* at genus level. Significance of each bacteria taxa: * *p* < 0.05; ** *p* < 0.01.

Procrustean analysis confirmed a strong relationship between the structure of the *phoD* gene community at Operational Taxonomic Unit (OTU) level and soil P fractions across treatment type (Fig. 6a). However, soil P fractions were not affected by the composition of the *phoX* gene community (Fig. 6b).

We next conducted a correlation analysis between the *phoD* and *phoX* gene communities and soil P fractions. At the family level of the *phoD* gene community, relative abundances of Bradyrhizobiaceae, Nocardiaceae, and Sphingomonadaceae were positively correlated with resin-Pi, NaHCO3-Pi, NaHCO_3_-Po, NaOH-Pi, and NaOH-Po. Meanwhile, Burkholderiaceae was negatively correlated with all P fractions except NaHCO_3_-Po (Fig. 6c). Spearman’s correlation analysis revealed a significant positive correlation between NaHCO_3_-Po and Methylobacteriaceae, conc. HCl-Pi with Oxalobacteraceae, Acetobacteraceae, and Methylobacteriaceae, and residual-P with Bradyrhizobiaceae, Sphingomonadaceae, and Methylobacteriaceae (Fig. 6c). At the genus level of the *phoD* community, the relative abundance of *Bradyrhizobium* and *Rhodococcus* was positively correlated with resin-Pi, NaHCO_3_-Pi, NaHCO_3_-Po, NaOH-Pi, and NaOH-Po. *Bradyrhizobium* was also positively correlated with residual-P (Fig. 6e). Further, Methylobacterium was positively correlated with resin-Pi, NaHCO_3_-Po, conc. HCl-Pi, and residual-P (Fig. 6e). In contrast, the relative abundance of *Mesorhizobium* was negatively correlated with NaHCO_3_-Po and conc. HCl-Pi (Fig. 6e).

## Discussion

Compared to drought, irrigation increased TP and TPi due to significant increases in labile and moderately labile P. Labile P, which serves as the primary source of P for plant growth, increased substantially by 2.34 fold (Table 2)^[35,36]^. However, labile P only accounted for 3% of TP under the irrigation treatment (Fig. 1a), indicating that available P deficiency was still an important limiting factor in this ecosystem's productivity. Nevertheless, improving soil moisture increased litter production and soil coverage, thereby reducing Pi leaching from the soil surface^[37]^. Additionally, higher litter input and the proliferation of roots have been shown to increase Po, and the partial decomposition of Po by plants and microorganisms can increase the availability of resin P^[38,39]^. NaHCO_3_-P increased significantly under irrigation and altered labile P composition, with NaHCO_3_-Pi gradually coming to dominate this fraction (Fig. 1b). Activated soil phosphatase promoted the release of inorganic P from NaHCO_3_-Po, which represents a labile form of Po that can rapidly dissolve and mineralize. These characteristics act to supplement P in deficient soils, mitigating declines in resin-Pi and NaHCO_3_-Pi.

NaOH-P is a component of moderately labile P pool in soils and requires long-term mineralization before it is available to plants^[40]^. Its presence therefore reflects the soil's future potential to supply P. Irrigation can increase NaOH-P content, likely as a result of the influx of external sources of carbon^[38,41]^. P represented by HCl-P is associated with calcium and is likely derived from primary minerals^[42,43]^. We found that 1 M HCl-Pi was the predominant form at our study location, accounting for approximately 70% of TP (Fig. 1c). Recent studies have shown that the mean residence time of HCl-P can range from years to millennia and that the average turnover rate of Ca-phosphate is 0.00088 g·g^−1^·d^−1^^[44,45]^, indicating that it is highly stable. However, it should be noted that the stability of HCl-P is significantly influenced by the soil pH^[45]^. In alkaline soils, the HCl-P pool is typically comprised of highly stable calcium-P minerals. Long-term irrigation can lead to increased soil moisture and decreased pH (Supplemental Fig. S1) and may facilitate the weathering of primary minerals and the desorption of Ca-associated P. Residual P, bound by secondary minerals, represents the most stable P fraction in soils. Although it is typically unavailable to plants and soil microorganisms, desorption and weathering can eventually mobilize it for plant uptake^[46]^.

The mineralization of Po to Pi is believed to be heavily influenced by phosphatases^[47,48]^. A substantial increase in both ACP and ALP activities in response to irrigation was observed in our study (Fig. 2a & b) and may be linked to increased litter input and higher soil water content. Irrigation stimulates enzyme activity because water increases the connectivity between soil pores and the availability of nutrient resources to meet the physiological needs of microorganisms^[6]^. Soil microbial biomass is increasingly recognized as a critical driver of soil P dynamics and we found a positive correlation between soil MBP and most soil P fractions (Fig. 3)^[49,50]^. Furthermore, irrigation increased MBP (Fig. 2c), while drought has been shown to inhibit microbial growth and cause the release of significant quantities of P, resulting in a reduction of MBP^[51]^.

Irrigation affected several soil microbiome characteristics, including microbial biomass, taxonomy, and functional profiles. Irrigation has been shown to increase both total and active microbial biomass in soil, as well as bacterial and fungal diversity (Fig. 4a & b). This suggests that adequate water availability facilitates the metabolic activities of soil microorganisms, promoting their growth and propagation. Moreover, water availability can influence soil microbial function^[52,53]^. Irrigation increased the number of *phoD* and *phoX* bacteria (Fig. 4c) and the diversity of *phoD* (Supplemental Fig. S2a & b), a gene encoding an important phosphatase enzyme involved in P cycling. This indicates that water availability enhances the ability of soil microbes to perform essential functions, such as nutrient cycling. Therefore, irrigation can significantly impact soil microbial function, ultimately affecting the overall health of soil ecosystems.

We also found that the composition and function of microorganisms, rather than total microbial biomass, significantly influenced soil P fractions, as demonstrated by variations in the diversity of bacteria and fungi and the copy numbers of *phoD* and *phoX* bacteria, which is involved in P mineralization (Fig. 5). Furthermore, a notable increase in the relative abundance of phosphorus solubilizing bacteria ([PSB] Bradyrhizobiaceae, Nocardiaceae, Sphingomonadaceae, Phyllobacteriaceae and Xanthomonadaceae) under irrigation was observed (Supplemental Fig. S3). Bradyrhizobiaceae and Sphingomonadaceae were also positively correlated with various P forms, including resin-Pi, NaHCO_3_-Pi, NaHCO_3_-Po, NaOH-Pi, and NaOH-Po (Fig. 6c). Bradyrhizobiaceae, which belong to the Proteobacteria phylum, are known to release organic acids that help solubilize different forms of P^[54]^, improving plant growth by increasing P availability in nutrient-poor forest soils^[55]^. Similarly, Sphingomonadaceae have been found to have a beneficial connection with ALP activity in phosphite-treated soil^[56]^. It is also known that Xanthomonadaceae promote organic P mineralization^[57,58]^. A significant positive correlation between NaHCO_3_-Po and Methylobacteriaceae was also found. *Methylobacterium* are copiotrophic bacteria that are widespread in nutrient-rich environments and are known to be correlated with SOC^[59,60]^. Due to its capacity to tolerate hostile environments, *Bacillus* is among the most abundant PSB categories^[61,62]^. However, the relative abundance of *Bacillus* in our study was uncorrelated with soil P fractions and did not significantly vary between irrigation and drought treatments. These results suggest that the irrigation status of soil had little effect on *Bacillus*, likely due to its high drought tolerance.

## Conclusions

This field study provided empirical evidence that irrigating *P. euphratica* plantations increase soil P availability and supply capacity and causes significant reallocation within soil P fractions. The enhanced mineralization of organic P was linked to variations in soil moisture and pH and to changes in the composition and functional profiles of soil microorganisms, mainly bacteria possessing *phoD* genes. However, it will be necessary to fully characterize the allocation of foliar-P fractions of *P. euphratica* and its relationship with soil-P fractions in the future. These findings underscore the potential impacts of water management on soil P dynamics.

## SUPPLEMENTARY DATA

Supplementary data to this article can be found online.
