# Supplementary material for: Effects of long-term irrigation on soil phosphorus fractions and microbial communities in Populus euphratica plantations
Source: For Res (Fayettev). 2023 Jul 26;3:17. doi: 10.48130/FR-2023-0017 (PMC11524274; doi:10.48130/FR-2023-0017)
Supplement: Supplementary file 1 — Supplementary data to this article can be found online. [file FR-2023-0017-S1.zip › 10.48130_FR-2023-0017-Suppl-FigureS2.docx]

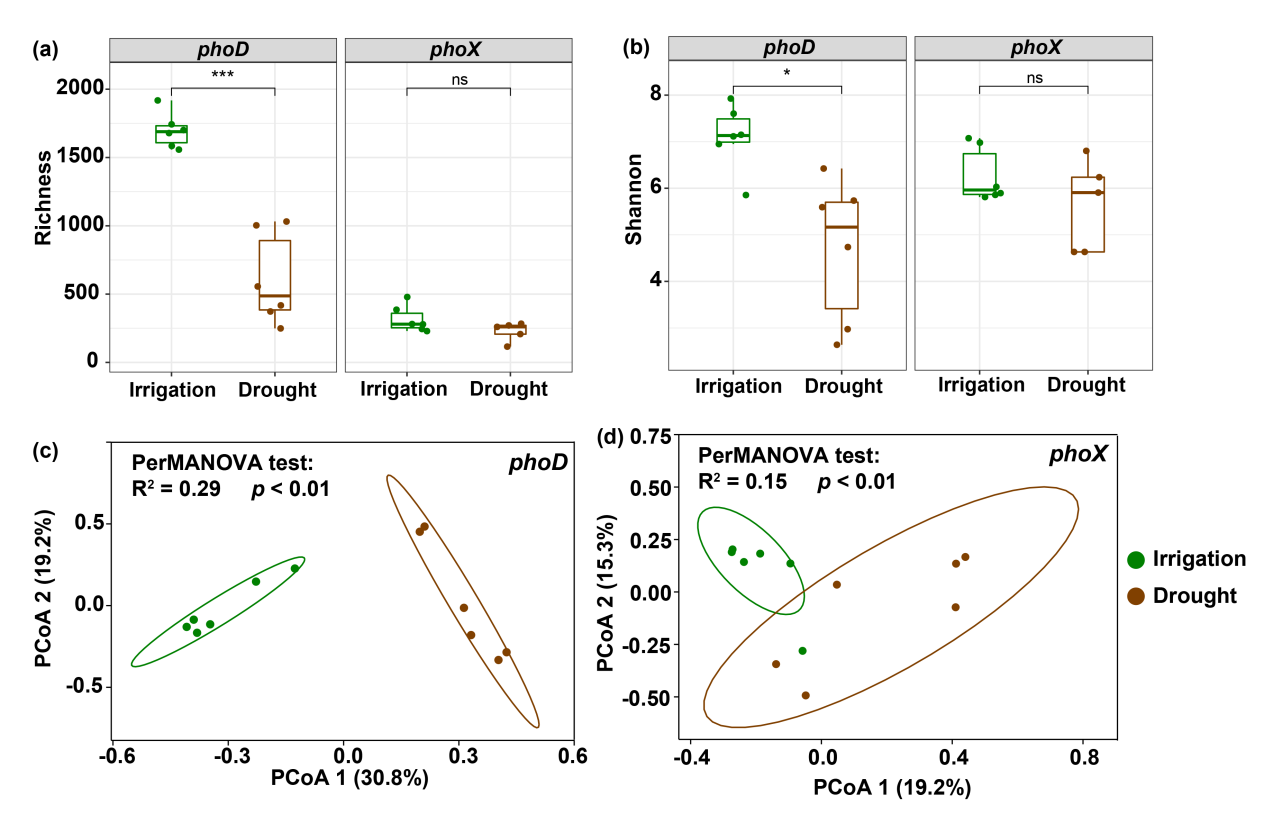


Figure S2 Comparisons of alpha and beta diversity of alkaline phosphatase gene communities under different water management treatments. (a) Richness of *phoD* and *phoX*. (b) Shannon-diversity of *phoD* and *phoX*. (c) Principal coordination analysis (PCoA) of *phoD*. (d) PCoA of *phoX.* Values on PCoA axes indicate the percentages of total variation explained by each axis.
