# Supplementary material for: Effects of long-term irrigation on soil phosphorus fractions and microbial communities in Populus euphratica plantations
Source: For Res (Fayettev). 2023 Jul 26;3:17. doi: 10.48130/FR-2023-0017 (PMC11524274; doi:10.48130/FR-2023-0017)
Supplement: Supplementary file 1 — Supplementary data to this article can be found online. [file FR-2023-0017-S1.zip › 10.48130_FR-2023-0017-Suppl-FigureS3.docx]

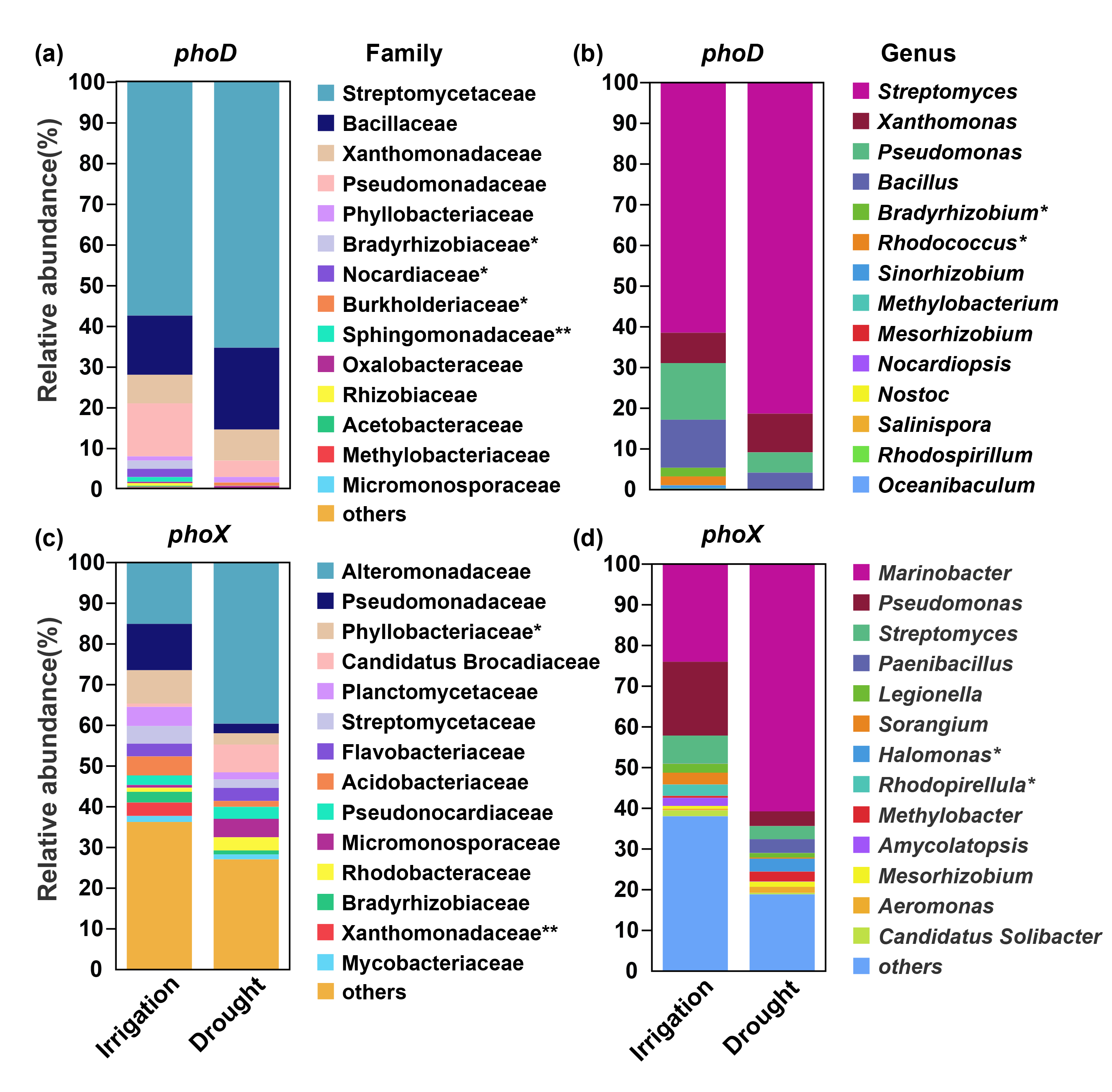


Figure S3 Relative abundance of dominant alkaline phosphatase gene communities (*phoD* and *phoX*) at the family and genus level under different water management treatments. (a) The relative abundance of *phoD* at the family level. (b) The relative abundance of *phoD* at the genus level. (c) The relative abundance of *phoX* at the family level. (d) The relative abundance of *phoX* at the genus level. Significance is indicated by **, *p* < 0.01; *, *p* < 0.05.
