# Supplementary material for: Effects of long-term irrigation on soil phosphorus fractions and microbial communities in Populus euphratica plantations
Source: For Res (Fayettev). 2023 Jul 26;3:17. doi: 10.48130/FR-2023-0017 (PMC11524274; doi:10.48130/FR-2023-0017)
Supplement: Supplementary file 1 — Supplementary data to this article can be found online. [file FR-2023-0017-S1.zip › 10.48130_FR-2023-0017-Suppl-Material-S1.docx]

**Supplementary material S1**

The extracted DNA concentrations were detected with a NanoDrop spectrophotometer (Thermo Fisher Scientific, Dreieich, Germany). The primers utilized for bacterial 16S (V4-V5) and fungal ITS rRNA gene amplifications were 338F: ACTCCTACGGGAGGCAGCAG and 806R: GGACTACHVGGGTWTCTAAT, and ITS3F: GCATCGATGAAGAACGCAGC and ITS4R: TCCTCCGCTTATTGATATGC, respectively. Bacterial and fungal amplicon libraries were prepared according to Quast *et al.*^[1]^ and Kõljalg *et al.*^[2]^.

Quantitative Polymerase Chain Reaction (qPCR) was used to measure *phoD* and *phoX* gene abundances, and amplicon sequencing was also conducted for *phoD* and *phoX*. For *phoD* and *phoX* gene abundances, the primer sets *phoD*-F733 (5′-TGGGAYGATCAYGARGT-3′) and *phoD*-R1083 (5′-CTGSGCSAKSACRTTCCA-3′) were used to amplify the *phoD* gene, while *phoX*-F455 (5′-CAGTTCGGBTWCAACAACGA-3′) and *phoX*-R1076 (5′-CGGCCCAGSGCRGTGYGYTT-3′) were used to amplify the *phoX* gene, as described by Ragot *et al.*^[3]^ and Chen *et al.*^[4]^. The qPCR reaction mixture (10 μl) was identical for both the *phoD* and *phoX* genes, and contained 5 μl of SsoFastTM EVaGreen® Supermix (Bio-Rad Laboratories Inc., CA, USA), 0.5 μl of each primer (10 μM), 1 μl of DNA template (10 ng μl^–1^) and 3.5 μL ddH_2_O. The thermocycler was programmed with an initial denaturation step at 98 ℃ for 2 min, followed by 36 cycles of 98 ℃ for 10 s, 59 ℃ for 1 min and 72 ℃ for 1 min. The qPCR standard curves had R^2^ values larger than 0.998, and the amplification efficiency exceeded 90%. PCR reactions were carried out in triplicate. The products from PCR were sequenced using the Illumina Nova 6000 PE250 platform (Illumina Inc., CA, USA) by Guangdong Magigene Biotechnology (Guangzhou, China).

Raw FASTQ files were quality-filtered by Trimmomatic and merged by FLASH^[5]^ with the default parameters. The optimized sequences were organized into operational taxonomic units (OTUs) using UPARSE 7.1 with a 97% sequence similarity threshold. The Silva (v132) database, UNITE (v8.0) database, *phoD* and *phoX* FunGene databases (v1.0, provided by Guangdong Magigene Biotechnology) were used to classify bacterial taxa, fungal taxa, and phosphate-solubilizing bacterial taxa identified from amplicon sequencing.
