# Supplementary material for: Effects of long-term irrigation on soil phosphorus fractions and microbial communities in Populus euphratica plantations
Source: For Res (Fayettev). 2023 Jul 26;3:17. doi: 10.48130/FR-2023-0017 (PMC11524274; doi:10.48130/FR-2023-0017)
Supplement: Supplementary file 1 — Supplementary data to this article can be found online. [file FR-2023-0017-S1.zip › 10.48130_FR-2023-0017-Suppl-FigureS1.docx]

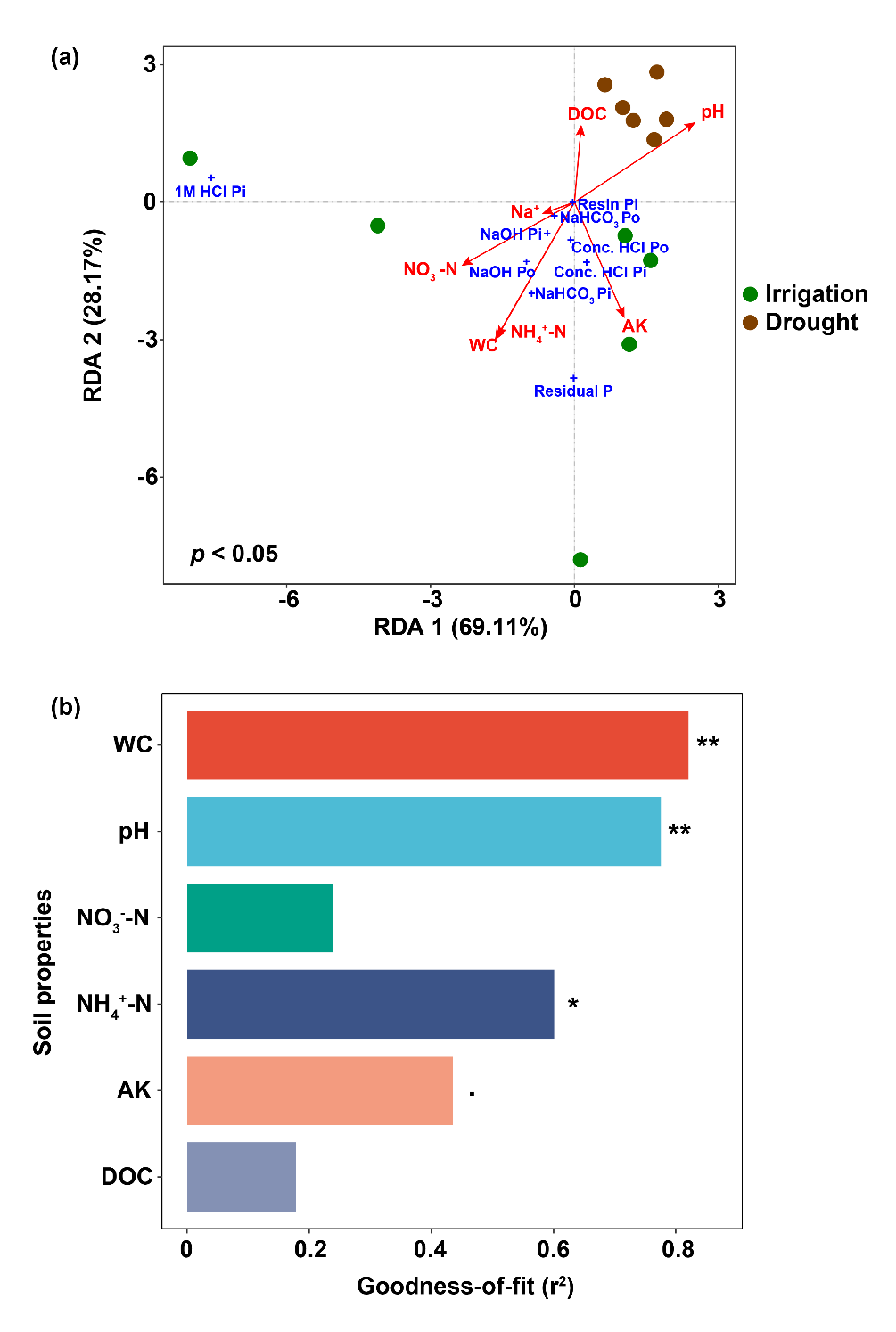


Figure S1 Redundancy analysis of soil P fractions impacted by soil properties. (a) RDA across all experimental units. (b) The variation in soil properties explaining soil P fractions. Red arrows represent soil properties. Blue crosses represent soil P fractions. Significance is indicated by **, *p* < 0.01; *, *p* < 0.05.
